# Supplementary material for: Crystal structure of an assembly intermediate of respiratory Complex II
Source: Nat Commun. 2018 Jan 18;9:274. doi: 10.1038/s41467-017-02713-8 (PMC5773532; doi:10.1038/s41467-017-02713-8)
Supplement: Supplementary file 1 — Supplementary Information [file 41467_2017_2713_MOESM1_ESM.pdf]

Supplementary Table 1. Primer sequences used for generating the FrdA$^{F116C/G392C}$ disulfide-trapped variant.

**FrdA$^{F116C/G392C}$**	
**F116C**	
Forward Primer	**TgC**GGCGGCATGAAAATCGAG
Reverse Primer	GCGACGTACGTTGACG
**G392C**	
Forward Primer	**tGc**TCTAACTCCCTGGCGG
Reverse Primer	CAGACGGTTTGCACCGTG

Supplementary Figure 1.

Chain	Salt Bridges	Hydrogen bonds	Non-bonded contacts
AB	1	9	98
CD	1	9	90

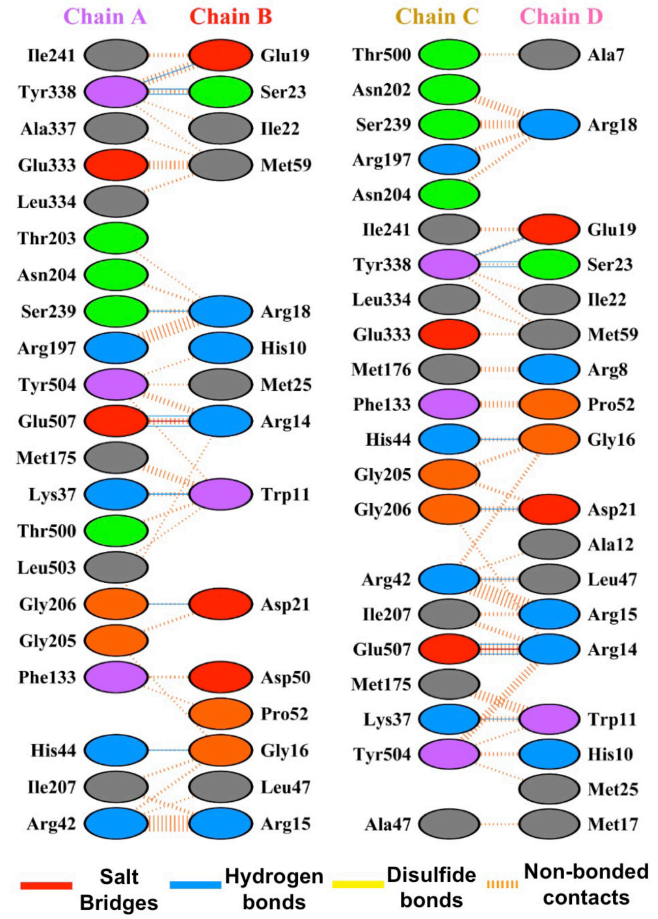

Supplementary Figure 1. Contacts between FrdA and SdhE. A schematic depiction of the contacts between FrdA and SdhE were analyzed using PDBsum server[1]; each of the two complexes in the asymmetric unit is shown in a different column. Salt bridges are shown as red lines (one per complex) hydrogen bonds are shown as blue lines (nine per complex), non-bonded contacts are shown as orange dashes (90 or 98, depending on the complex). There are no disulfide bonds. Contacts in the left column are for the complex containing the A and B chains, while those in the right column are for the complex containing the C and D chains. Neutral side chains are depicted as grey ovals, polar are green ovals, aromatic are magenta ovals, positively charged are blue ovals, and negatively charged are red ovals.

Supplementary Figure 2.

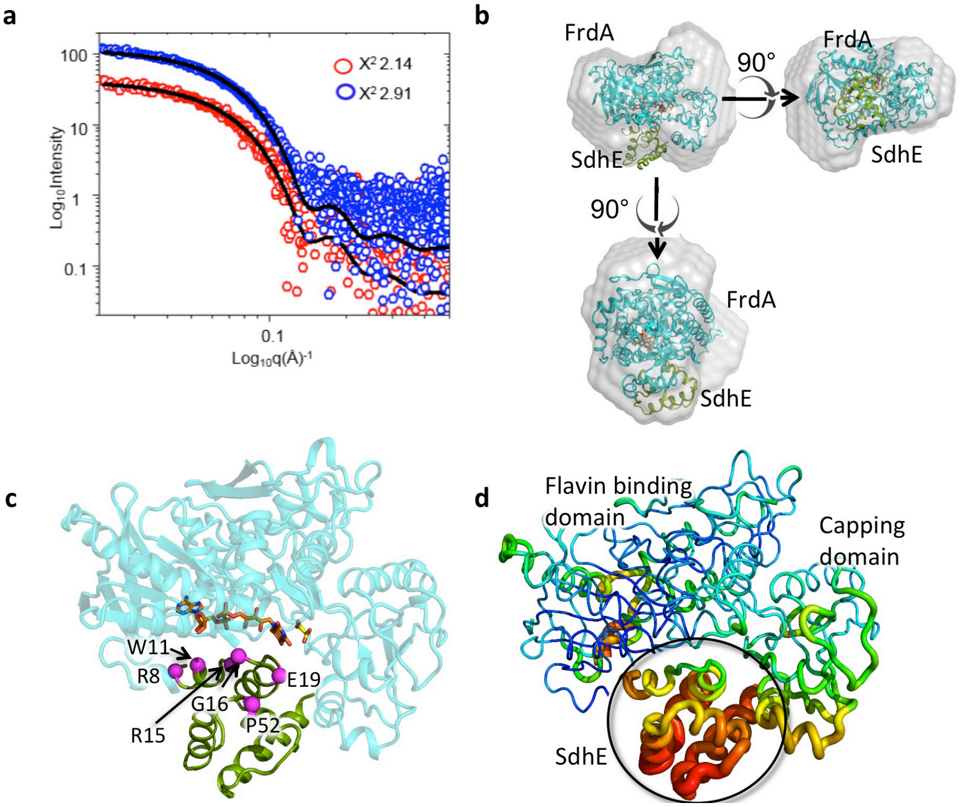

Supplementary Figure 2. Validation of the global structure. a. Comparison of the theoretical scattering curve (black line) of the FrdA-SdhE assembly intermediate with experimental scattering curves of SAXS data from two protein concentrations[2]. The χ^2 values of 2.14 and 2.91 suggest a reasonable correlation between the crystal structure and the structure in solution. b. The crystal structure of the FrdA-SdhE assembly intermediate superimposed onto the experimental SAXS envelope[2]. c. Positions of SdhE missense mutations that reduce covalent flavinylation of FrdA/SdhA subunits mapped onto the structure of the FrdA-SdhE assembly intermediate; a short region of FrdA that would obscure the binding interface in this view has been omitted for clarity. Mutagenesis of residues analogous to *E. coli* SdhE^{R8}, SdhE^{W11}, SdhE^{R15}, SdhE^{E19}, and SdhE^{P52} each reduce covalent flavinylation of their cognate flavoprotein[3,4]. These residues map to the interface of the FrdA-SdhE complex, suggesting that these missense mutations disrupt complex formation. The binding mode observed in the crystal structure further explains how the reported $\mathrm{SdhAF2}^{G78R}$ human missense mutation could result in Complex II deficiency and disease[5], with analogous mutations in yeast and bacteria eliminating covalent flavinylation in those systems[4,5]. $\mathrm{SdhAF2}^{G78}$ is equivalent to *E. coli*, SdhE^{G16}, which hydrogen-bonds and orients the side chain of the histidyl ligand, FrdA^{H44}. d. Crystallographic temperature factors of the FrdA-SdhE assembly intermediate mapped onto the structure. The SdhE subunit has elevated temperature factors, likely reflecting mobility or a dynamic interface.

Supplementary Figure 3.

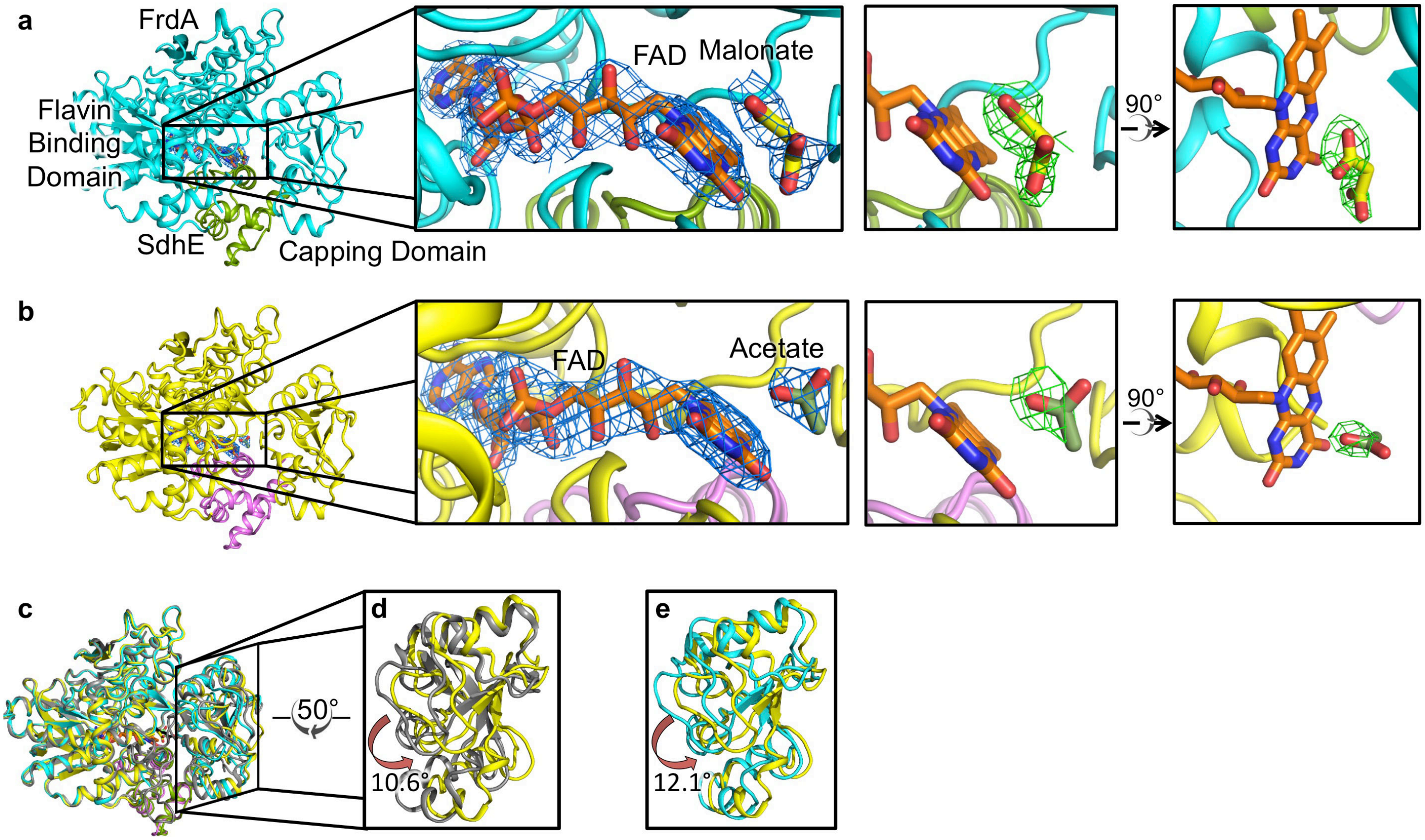

Supplementary Figure 3. Comparison of acetate-bound and malonate-bound FrdA-SdhE complexes. a. Active site of the malonate-bound FrdA-SdhE assembly intermediate superimposed onto $2F_o - F_c$ maps calculated following the removal of FAD and malonate from the PDB coordinates and contoured at 1 σ. The panels at right show two views of malonate superimposed onto $F_o - F_c$ omit maps calculated after removal of malonate from the PDB coordinates and contoured at 2.5 σ. b. Active site of the acetate-bound FrdA-SdhE assembly intermediate superimposed onto $2F_o - F_c$ omit maps and contoured at 1 σ. The panels at right show two views of acetate superimposed onto $F_o - F_c$ omit maps calculated after removal of acetate from the PDB coordinates and contoured at 2.5 σ. c. Comparisons of capping domain position between the acetate-bound FrdA-SdhE assembly intermediate (*yellow/pink*), fumarate-bound FrdABCD (*grey*; PDB entry 3P4P[6]) and the malonate-bound FrdA-SdhE assembly intermediate (*cyan/green*). d. Comparison of between the acetate-bound FrdA-SdhE assembly intermediate (*yellow*), fumarate-bound FrdABCD (*grey*; PDB entry 3P4P[6]) e. Comparison of the acetate-bound FrdA-SdhE assembly intermediate (*yellow*), and the malonate-bound FrdA-SdhE assembly intermediate (*cyan*).

Supplementary Figure 4.

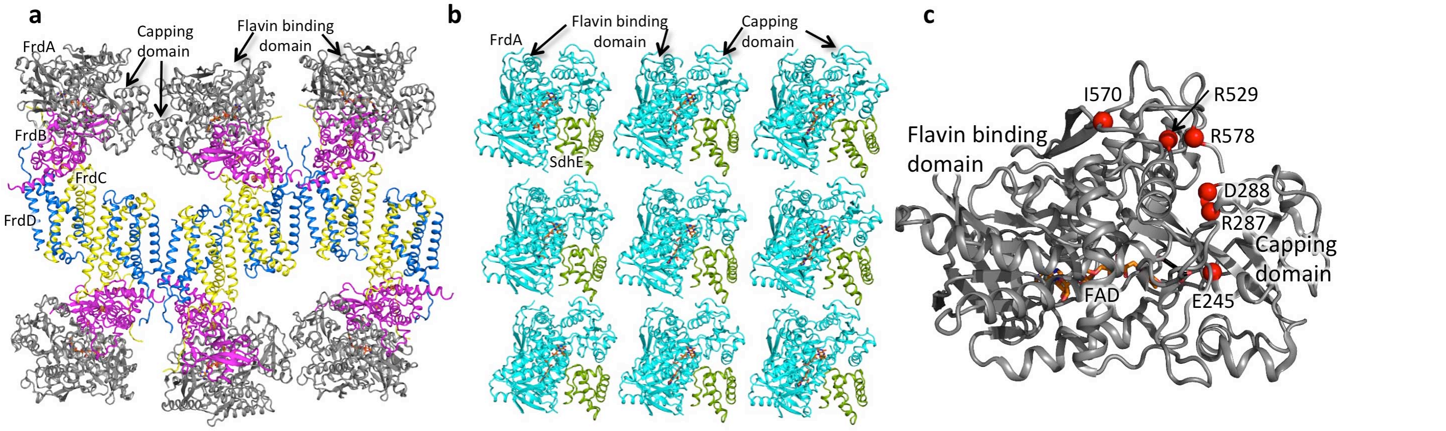

Supplementary Figure 4. Capping domain analyses. a and b. Crystal packing contacts stabilize the position of the capping domain. a. Crystal packing contacts of the intact FrdABCD complex (PDB entry 3P4P[6]); FrdA (grey), FrdB (magenta), FrdC (yellow), FrdD (blue). b. Crystal packing contacts of the FrdA-SdhE assembly intermediate; FrdA (cyan), SdhE (green). c. Locations of missense mutations of FrdA that result in loss of covalent flavinylation. We propose that $FrdA^{E245}$, $FrdA^{R287}$, $FrdA^{H355}$, and $FrdA^{R390}$ are directly involved in the chemistry of covalent flavinylation, as described in the main text. In contrast, the remaining mutations may effect capping domain position. These additional mutations that affect covalent flavinylation were identified in yeast and include yeast $Sdh1^{C630}$/$Sdh1^{R638}$ (equivalent to *E. coli* $FrdA^{I570}$/$FrdA^{R578}$), which are located in the distal C-terminus. Residues equivalent to $FrdA^{R578}$ (flavin-binding domain) and $FrdA^{D288}$ (capping domain) interact in crystal structures of FrdABCD and SdhABCD from multiple organisms[7-12], identifying this as a conserved interdomain contact. While $FrdA^{R578}$ is disordered in most structures of *E. coli* FrdABCD and the FrdA-SdhE structure presented here, truncation of this region in *E. coli* FrdA ($FrdA^{\Delta C576}$) results in loss of detectable assembly of FrdABCD under the conditions we tested (Iverson, unpublished). Similarly anticipated to control domain orientation in a substrate-independent manner, the conserved yeast $Sdh1^{R582}$ (*E. coli* $FrdA^{R529}$) is located near the interface of the flavin-binding domain and capping domain, where perturbation of the local structure via mutation may alter domain position. Missense mutation of the analogous residue in humans ($SdhA^{R589}$) is associated with abdominal paraganglioma[13].

Supplementary Figure 5.

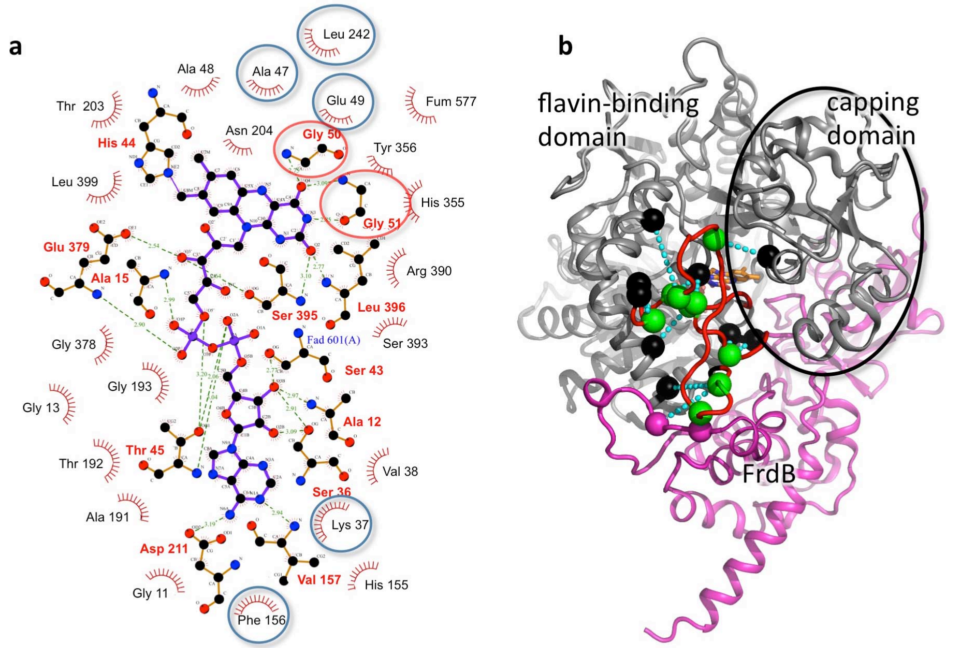

Supplementary Figure 5. Effect of SdhE on FrdA a. LigPlot[14] analysis of the contacts between FAD and protein. Contact between FrdA and FAD that are observed in both the FrdA-SdhE assembly intermediate and the FrdABCD complex are not circled; contacts found in FrdABCD but absent in FrdA-SdhE assembly are circled in red (hydrogen bonds) and blue (non-banded contacts). b. Analysis of hydrogen-bonding contacts for the loops (50-58 and 103-129) of FrdA in the context of the mature FrdABCD complex. Interacting residues from the loop regions are highlighted in green, and the destination interactions are shown as black (FrdA subunit) or magenta (FrdB subunit). Interactions are indicated with dashed lines (cyan). Rotation of the capping domain is anticipated to remove one hydrogen-bonding interaction, and lack of the FrdB Fe:S subunit is anticipated to remove two hydrogen-bonding interactions. Hydrophobic interactions also stabilize the position of these loops.

Supplementary Figure 6.

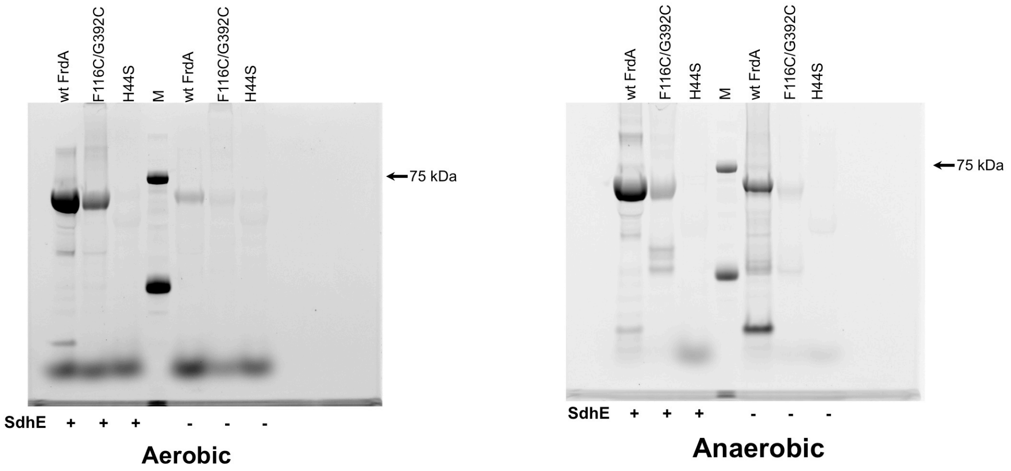

Supplementary Figure 6. Full gels showing the assessment of covalent flavinylation in wild-type FrdA and its variants. FAD fluorescence was monitored by running equivalent amounts of wild type FrdA, FrdA$^{F116C/G392C}$ and FrdA^{H44S} (negative control) proteins on SDS-PAGE. Reduced levels of covalent flavinylation was observed for FrdA$^{F116C/G392C}$ variant as compared to wild type FrdA.

Supplementary Figure 7.

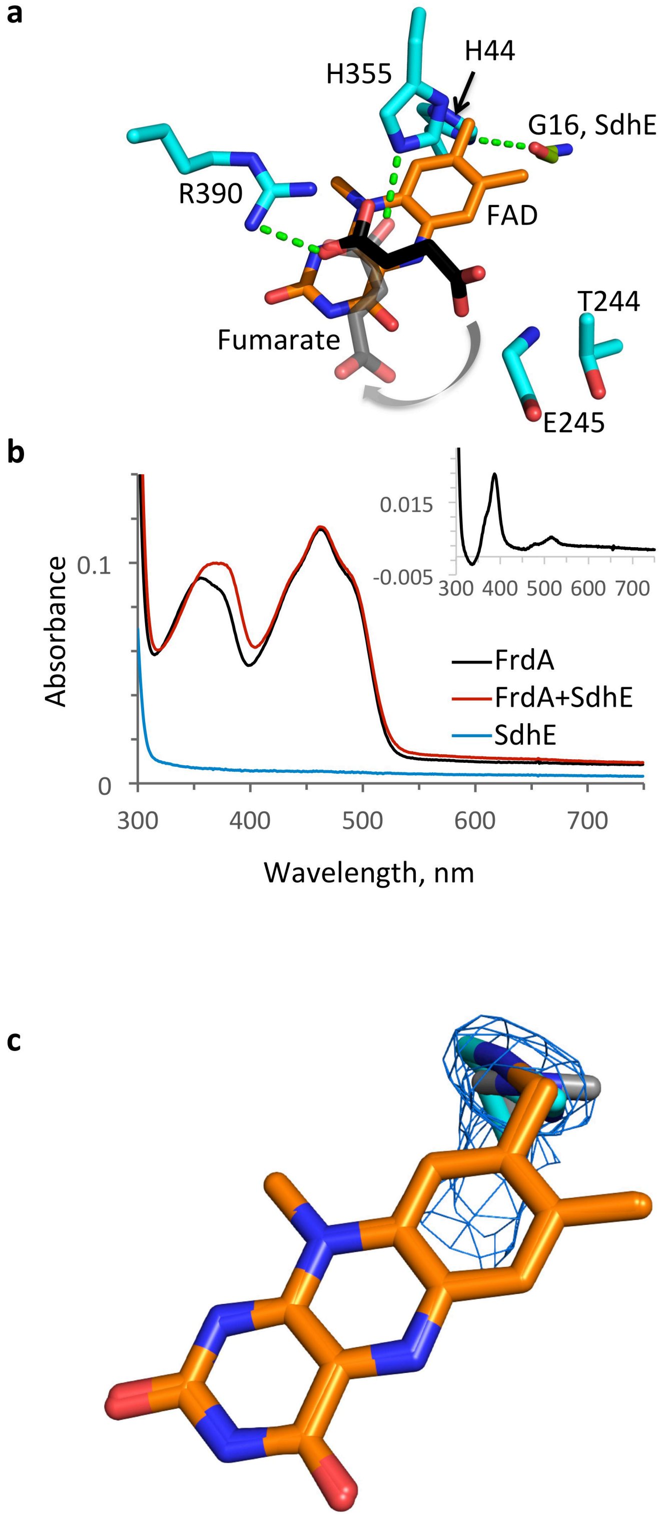

Supplementary Figure 7. Mechanism. a. Based upon the model of FrdA-SdhE assembly intermediate with malonate, fumarate is modeled into the active site (transparent bonds). This modeled position is nominally rotated 54° from the position in the experimental structure of FrdABCD with fumarate (PDB entry 3P4P, solid bonds), but additionally flips the molecule based upon the malonate alignment. In this mode, the C2=C3 bond is no longer near the N5 of FAD, preventing facile transfer of hydride from FAD N5. b. The K_d^{SdhE} for the FrdA subunit, as measured by optical difference spectroscopy. The FrdA-SdhE structure suggests that SdhE promotes changes the environment of the isoalloxazine ring of FAD, as a result we measured K_d^{SdhE} using optical

spectroscopy, which informs on isoalloxazine environment. The spectrum of isolated FrdA subunits (*black line*) exhibit a typical spectrum for FAD bound to Complex II. We find that the addition of SdhE (*red line*) promotes a red spectral shift in the high-energy 370 nm peak of FAD, while isolated SdhE (*blue line*) exhibits no detectable signal. We used this method of monitoring the interactions between FrdA or SdhA subunits and SdhE to measure the K_d^{SdhE} values, as reported in Table 1. c. Superposition of FAD and FrdA^{H44} onto $2F_o - F_c$ maps contoured at 2 σ. The orientation of the FrdA^{H44} imidazole ring from the FrdA-SdhE assembly intermediate (*cyan*) explains the electron density while the orientation of the FrdA^{H44} imidazole ring from the assembled FrdABCD complex (*grey*) is inconsistent with this electron density.

Supplementary References

1 Laskowski, R. A. *et al.* PDBsum: a Web-based database of summaries and analyses of all PDB structures. *Trends Biochem Sci* **22**, 488-490 (1997).

2 Starbird, C. A. *et al.* Structural and biochemical analyses reveal insights into covalent flavinylation of the Escherichia coli Complex II homolog quinol:fumarate reductase. *The Journal of biological chemistry* **292**, 12921-12933, doi:10.1074/jbc.M117.795120 (2017).

3 Eletsky, A. *et al.* Solution NMR structure of yeast succinate dehydrogenase flavinylation factor Sdh5 reveals a putative Sdh1 binding site. *Biochemistry* **51**, 8475-8477, doi:10.1021/bi301171u (2012).

4 McNeil, M. B. & Fineran, P. C. The conserved RGxxE motif of the bacterial FAD assembly factor SdhE is required for succinate dehydrogenase flavinylation and activity. *Biochemistry* **52**, 7628-7640, doi:10.1021/bi401006a (2013).

5 Hao, H. X. *et al.* SDH5, a gene required for flavination of succinate dehydrogenase, is mutated in paraganglioma. *Science* **325**, 1139-1142, doi:10.1126/science.1175689 (2009).

6 Tomasiak, T. M. *et al.* Geometric restraint drives on- and off-pathway catalysis by the Escherichia coli menaquinol:fumarate reductase. *The Journal of biological chemistry* **286**, 3047-3056, doi:10.1074/jbc.M110.192849 (2011).

7 Starbird, C. A. *et al.* Structural and biochemical analyses reveal insights into covalent flavinylation of the Escherichia coli Complex II homolog quinol:fumarate reductase. *J Biol Chem*, doi:10.1074/jbc.M117.795120 (2017).

8 Huang, L. S. *et al.* 3-nitropropionic acid is a suicide inhibitor of mitochondrial respiration that, upon oxidation by complex II, forms a covalent adduct with a catalytic base arginine in the active site of the enzyme. *The Journal of biological chemistry* **281**, 5965-5972, doi:10.1074/jbc.M511270200 (2006).

9 Ruprecht, J., Yankovskaya, V., Maklashina, E., Iwata, S. & Cecchini, G. Structure of Escherichia coli succinate:quinone oxidoreductase with an occupied and empty quinone-binding site. *The Journal of biological chemistry* **284**, 29836-29846, doi:10.1074/jbc.M109.010058 (2009).

10 Shimizu, H. *et al.* Crystal structure of mitochondrial quinol-fumarate reductase from the parasitic nematode Ascaris suum. *J Biochem* **151**, 589-592, doi:10.1093/jb/mvs051 (2012).

11 Sun, F. *et al.* Crystal structure of mitochondrial respiratory membrane protein complex II. *Cell* **121**, 1043-1057, doi:10.1016/j.cell.2005.05.025 (2005).

12 Yankovskaya, V. *et al.* Architecture of succinate dehydrogenase and reactive oxygen species generation. *Science* **299**, 700-704, doi:10.1126/science.1079605 (2003).

13 Burnichon, N. *et al.* SDHA is a tumor suppressor gene causing paraganglioma. *Hum Mol Genet* **19**, 3011-3020, doi:10.1093/hmg/ddq206 (2010).

14 Wallace, A. C., Laskowski, R. A., Singh, J. & Thornton, J. M. Molecular recognition by proteins: protein-ligand interactions from a structural perspective. *Biochem Soc Trans* **24**, 280-284 (1996).
