# Supplementary material for: Crystal structure of an assembly intermediate of respiratory Complex II
Source: Nat Commun. 2018 Jan 18;9:274. doi: 10.1038/s41467-017-02713-8 (PMC5773532; doi:10.1038/s41467-017-02713-8)
Supplement: Supplementary file 3 — Description of Additional Supplementary Files [file 41467_2017_2713_MOESM3_ESM.pdf]

**Description of Supplementary Files**

File Name: Supplementary Movie 1
Description: **View of the FrdA$^{H44}$-FAD covalent bond in the FrdA-SdhE assembly intermediate.** The movie overlays the covalent bond between FrdA$^{H44}$ and the FAD C8α in the FrdA-SdhE complex (cyan) as compared to the FrdABCD complex (grey). Rotation allows the angles to be viewed.
